# Biochemical assessment of the nutritional status of infants, children and adolescents in South Africa (1997–2022): a systematic review

**DOI:** 10.1017/S136898002400137X

**Published:** 2024-10-21

**Authors:** Linda Malan, Lizelle Zandberg, Marina V Visser, Mariaan Wicks, H Salome Kruger, Mieke Faber

**Affiliations:** 1 Centre of Excellence for Nutrition, North-West University, Potchefstroom 2520, South Africa; 2 Medical Research Council Unit for Hypertension and Cardiovascular Disease, North-West University, Potchefstroom, South Africa; 3 Non-Communicable Diseases Research Unit, South African Medical Research Council, Cape Town, South Africa

**Keywords:** Micronutrient deficiencies, Inflammation, South Africa, Infants, Children, Adolescents, Nutritional status biomarkers

## Abstract

**Objective:**

To conduct a systematic review of the published peer-reviewed articles on the biochemical assessment of nutritional status of South African infants, children and adolescents in 1997–2022.

**Design:**

Online databases (Pubmed, CINAHL, EbscoHost and SAePublications) were used to identify thirty-nine papers.

**Setting:**

South Africa, 1997–2022.

**Participants:**

Infants, children and adolescents.

**Results:**

Vitamin A deficiency prevalence was 35–67 % before 2001 and mostly below 16 % after 2008. Anaemia ranged from 5·4 to 75·0 %, with 36–54 % of infants below 1 year being anaemic. Among 0- to 6-year-olds, iron deficiency (ID) was 7·2–39·4 % in rural and 16–41·9 % in urban areas. Zn deficiency remained high, especially among 0- to 6-year-olds, at 39–48 %. Iodine insufficiency (UIC < 100 µg/l) was between 0 and 28·8 %, with excessive levels in two areas. Vitamin D deficiency was 5 % for 11- to 17-year-olds in one urban study but 33–87 % in under 10-week-old infants. The 2005 national survey reported sufficient folate status among 0- to 6-year-olds, and vitamin B_12_ deficiency was 0–21 %. Low-grade inflammation was between 5 % and 42 % depending on the biomarker and cut-offs.

**Conclusions:**

Vitamin A status may have improved meaningfully during the last 25 years in South Africa to below 16 %, and iodine and folate deficiency appears to be low particularly among 0- to 6-year-olds. However, confirmation is needed by a national survey. Anaemia, Fe and Zn deficiencies still pose severe problems, especially among 0- to 6-year-olds. Sufficient data on vitamin D and B_12_ status are lacking.

## Introduction and rationale

Micronutrient deficiencies among infants, children and adolescents are a pressing public health concern in South Africa (SA) and are associated with stunting, increased morbidity, mortality, loss of developmental potential, poor educational performance, as well as increased risks of chronic diseases in adulthood^(1,2)^. The complementary diets of infants in SA are characterised by a low dietary diversity^(3,4)^, which contributes to micronutrient deficiencies during early childhood and typically persists into later childhood^(5)^.

Micronutrients such as Fe, Zn, iodine, vitamin A, D, B_12_ and folate are crucial for growth and development, particularly in infants and young children but also in older children subsequently leading to an intergenerational cycle of malnutrition^(5)^. During these phases of fast development, environmental insults may have irreversible negative effects^(6)^. Young children who have optimal nutritional status subsequently contribute more to the social and economic growth of their communities, as well as their own physical and mental development^(7)^. Iron deficiency (ID) and iron deficiency anaemia (IDA) are the most common nutritional disorders worldwide and one of the leading contributors to the global burden of disease^(8)^. Young children are at risk for ID and IDA because of their higher need for Fe. The WHO recommends considering inflammation in areas with widespread infection or inflammation when assessing changes in biomarkers of nutritional status. Therefore, data on C-reactive protein (CRP) and alpha-1 acid glycoprotein (AGP) and the prevalence of inflammation are included in this review^(9,10)^.

The South African government has introduced several strategies since 1997 to improve infant and young child nutrition, i.e. routine periodic high-dose vitamin A supplementation (VAS) according to WHO guidelines^(11,12)^, the Infant and Young Child Feeding Policy (2007, revised 2013), the Roadmap for Nutrition in SA (2012)^(4)^ and the National Integrated Early Childhood Development Policy (2015)^(13)^. Examples of strategies for improvement in school-age child nutrition include the National School Nutrition Programme^(14)^ and the Integrated School Health Policy (2012) which includes nutrition as one of the twelve issues covered^(15)^. In addition, the National Food Fortification Programme (NFFP) comprising vitamin A, Fe, Zn, folic acid, thiamine, niacin, vitamin B_6_ and riboflavin added to maize meal and wheat flour in SA since October 2003 contributes to nutrition at all stages of the lifecycle^(16)^. Nevertheless, most micronutrient deficiencies remain high, with possible improvement only seen for vitamin A deficiency^(17,18)^.

Previous national surveys in SA reported 64 % and 44 % vitamin A deficiency, 28 % and 11 % anaemia, 20 % and 8 % IDA, in 1- to 9-year-olds and children under five, respectively, and 45 % Zn deficiency in 1- to 9-year-olds^(19,20)^. In addition to national surveys, smaller regional studies also provide valuable information and are essential for informing policies and programmes to address region-specific nutritional status challenges. Biochemical nutritional status among healthy South African infants, children and adolescents has not been reviewed recently^(21)^.

This study aimed to conduct a comprehensive systematic review of all the published peer-reviewed articles on the biochemical assessment of the nutritional status of South African infants, children and adolescents from 1997 to 2022 and report on the prevalence and changes of micronutrient deficiencies during this period.

### Protocol

The study team used the Preferred Reporting Items for Systematic Reviews and Meta-analysis (PRISMA) guidelines to draft the protocol and then further refined it. Since this review had no health outcomes, the protocol was not registered with PROSPERO. It was outside the scope of this systematic review to identify literature gaps, as the focus was on prevalence and changes over time.

## Methods

### Eligibility criteria for study selection

Observational cross-sectional studies as well as the baseline data of randomised controlled trials or prospective studies published in English from 1997 to 2022 containing biochemical data on the nutritional status of South African infants, children and adolescents were included in this systematic review. The inclusion criteria were the following: healthy South African infants, children and adolescents, with original quantitative data on biochemical assessment of nutritional status. Endpoints of intervention studies were excluded. Studies were further excluded if they were clinical studies in patient subgroups, young pregnant or lactating women and/or particularly vulnerable groups. However, studies in large groups from low- socio-economic status were included, as a large proportion of South African infants, children and adolescents live in low socio-economic households. Narrative and systematic reviews, letters, editorials, case-control and qualitative studies, as well as studies with data collection before 1997 were excluded. Most of the latter were included in a previous review of the nutritional status of South African children^(21)^.

### Search strategy

Literature searches were performed in PubMed, Ebscohost, CINAHL and the South African ePub databases for the period 1 January 1997 to 31 July 2022, by using a structured search strategy based on the eligibility criteria. The search strategies were drafted by an experienced librarian from the North-West University and further refined through team discussion. Relevant keywords were identified from the Medical Subject Headings (MeSH) terms and adapted for each database. The search syntax for PubMed, Ebscohost and CINAHL is shown in Table 1. The syntax was modified for the African Journals database, with ‘nutrition’ in ‘Anywhere’ and filters for ‘Medicine and Health’, SciELO SA and the start and ending dates due to the limited options in the advanced search. We used an iterative process to identify appropriate search terms, including a term regarding biochemical nutritional status (anaemia, ID, Zn deficiency, vitamin A deficiency, iodine deficiency, vitamin D or micronutrient status) and children (terms for the different age groups). We also included ‘South Africa’ and date of publication in the search string. No grey literature was included, as most studies from South African students’ dissertations are published in South African scientific journals. Also, unpublished dissertations would probably not achieve the required quality score for inclusion in this review.

**Table 1:** Search terms for the literature search

#1:	All fields/All Text Fields	‘nutritional status’ OR malnutrition OR anemia OR anaemia OR ‘iron deficiency’ OR ‘zinc deficiency’ OR ‘vitamin A deficiency’ OR ‘vitamin D deficiency ‘OR ‘iodine deficiency’ OR ‘micronutrient status’
AND	Title/Abstract	‘South Africa’ OR ‘sub-Sahara Africa’
AND	Title/Abstract	infant OR baby OR child OR pediatric OR paediatric OR ‘young child’ OR toddler OR pre-school OR preschool OR ‘early childhood’ OR adolescent OR teenager OR youth
AND	Date – Publication/ Publication year	1997/01/01 to 2022/03/31 1997 to 2022

### Title, abstract and full-text screening and quality assessment

Titles and abstracts retrieved from electronic searches were screened by two independent reviewers (LM and LZ) after initial removal of duplicates. If the two reviewers could not agree on inclusion, they consulted with a third reviewer (HSK) and made a final decision based on consensus. Eligible studies were selected based on the inclusion and exclusion criteria. Finally, full-text articles were screened, and reasons for exclusion were noted. Reviews were excluded, but additional studies were identified from the reference lists of systematic and narrative reviews. Eligible studies were further screened by two independent reviewers (LM and LZ) for assessment of the quality of the reported data, based on the Joanna Briggs Institute critical appraisal scoring system for studies reporting prevalence data proposed by Munn^(22)^. The scoring tool includes questions on participant sampling, description, response rate, identification of the condition studied and data analysis. A ‘Yes’ answer to each question received a score of one, while a ‘No’ answer received a score of zero, with a maximum score of 9 (online Supplementary Table 1). A minimum total score of 5 was set as the threshold for final inclusion of a study into the systematic review. The time of data collection was obtained from the corresponding authors by email when not stated in the articles.

### Data extraction and synthesis

**Fig. 1:**
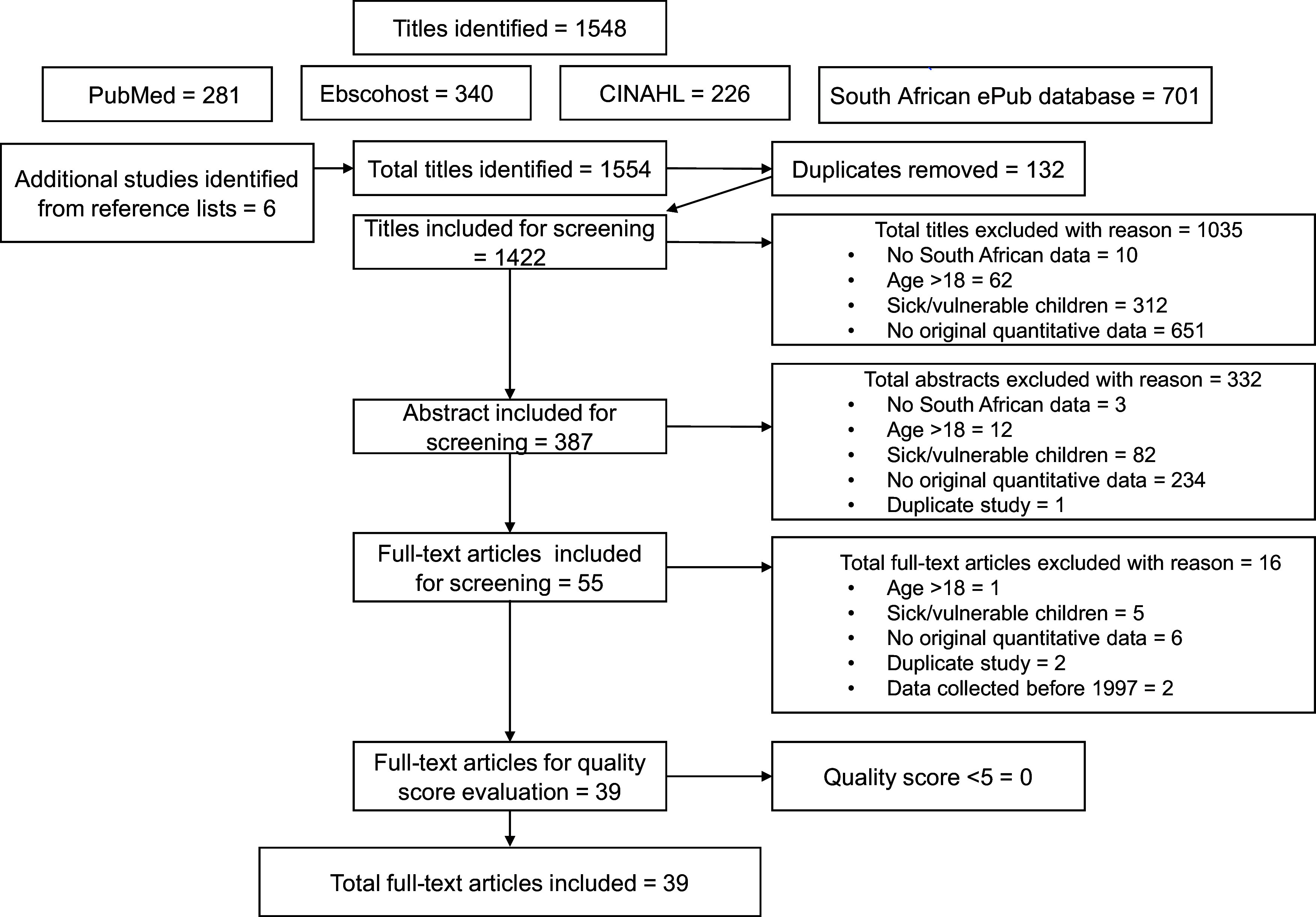
PRISMA flow diagram of the screening procedure followed to identify eligible studies

A flowchart showing the data extraction process is provided in Fig. 1. A data extraction form based on the review objectives was developed. Two reviewers piloted and finalised the form (LM and LZ). One reviewer recorded the data extracted from each eligible study (LM). A second reviewer (LZ) checked the extracted data, and in case of differences, the data were discussed with a third reviewer (HSK). The following information was extracted: (a) first author’s surname and publication date; (b) year of study (c) province where the study was conducted; (d) the study setting and location (rural or urban); (e) participants’ age range; (f) sample size; (g) mean ± sd or median and interquartile range of the biomarker, (h) prevalence (deficiency or inflammation) and (i) reference or cut-off points used to indicate nutritional status.

The biochemical indicators included for the respective nutrient status assessments were serum retinol for vitamin A status, Hb for anaemia, plasma/serum ferritin and serum transferrin receptor (TfR) for Fe status, serum Zn for Zn status, urinary iodine concentration for population iodine status, serum 25-hydroxy vitamin D (25(OH)D) as indicator for vitamin D status, serum and red blood cell folate and serum vitamin B_12_, as well CRP and AGP as markers of inflammation.

Data from studies on each deficiency and inflammation prevalence were summarised in tables according to age category, rural or urban settings, and province where the data were collected. Data were presented according to three age groups: infants and preschool children (0–6 years), primary school-age children (6–13 years) and adolescents (10–19 years). When the data were reported according to overlapping age groups, for example 10–14 years, the data were presented in the category representing most of the children. The prevalence data were also represented graphically over time, differentiated by age groups. Linear regressions were performed, and trend lines were indicated for all studies over time. Since national study data were weighted to ensure representativeness, but not regional study data, an additional regression was performed for national representative data alone. The slope depicts the change in percentage per year and *R* ^2^ indicates the proportion of the variance in the dependent variable (% deficiency) that is predictable from the independent variable (time in years). The prevalence of different nutritional status indicators per age category over time was compared to note any improvements or deterioration in biochemical nutritional status over the period of study (1997–2022).

## Results

In total, 1548 titles were identified, with an additional six articles identified from reference lists (Fig. 1). Of these, 387 abstracts and 55 full-text articles were screened for inclusion, with 39 full-text articles being assessed for quality, of which all were included. All articles scored greater than 5 out of 9 in terms of quality from both reviewers, and none were excluded (online Supplementary Table 1). Studies not designed to assess prevalence, such as randomised controlled trials or non-randomised control groups (baseline data) and some longitudinal studies scored negative for question 1, on representativeness of the target population and question 2, on sampling method (recruitment) not being random. The kappa statistic for agreement between the scores of two independent reviewers was 0·61, *P* < 0·001, and the intra-class correlation for single measures was 0·80 (*P* < 0·001). Kappa was interpreted using 0·21–0·40 as fair agreement, 0·41–0·60 as moderate and > 0·60 as very good agreement^(23)^. Intra-class correlation coefficients were interpreted as 0·50–0·75 as moderate and > 0·75 as good agreement^(24)^.

This review presents data on a total of thirty-nine studies, including three national surveys^(19,20,25)^, from all nine provinces of SA, with the sample sizes ranging from 39 to 1730 participants. Only one study included adolescents but did report the prevalence of deficiency^(26)^. Race and ethnicity of the study participants were not always reported. National studies and regional studies with large sample sizes generally included children from all race groups. Regional studies all pose a risk of bias due to being unrepresentative with mostly convenience sampling applied and small sample sizes. Nevertheless, they contribute to the body of evidence of micronutrient deficiencies in the country.

### Vitamin A status

**Fig. 2:**
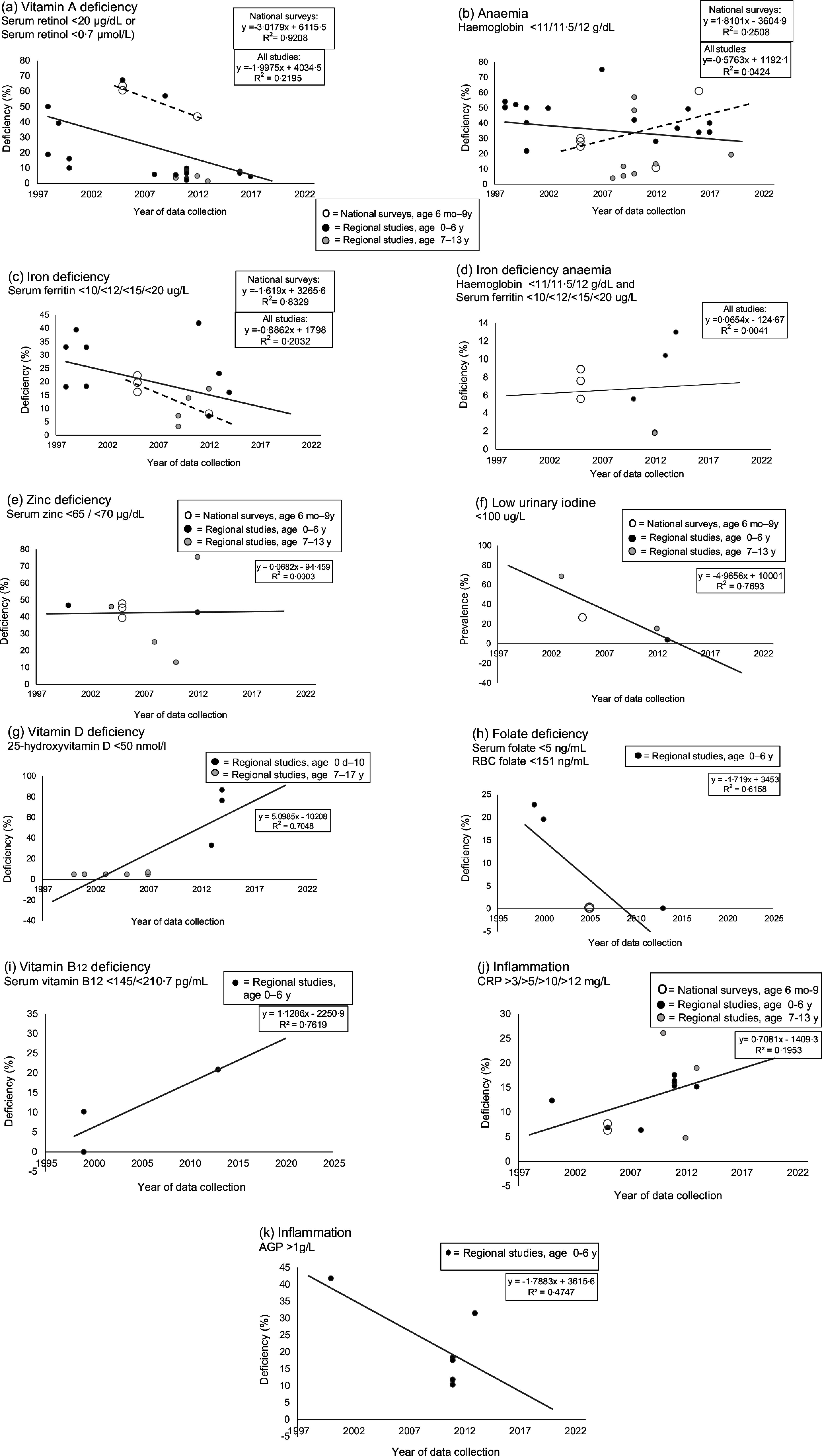
Graphical representation of (a) vitamin A deficiency, (b) anaemia, (c) iron deficiency, (d) iron deficiency anaemia, (e) Zn deficiency, (f) low urinary iodine, (g) vitamin D deficiency, (h) folate deficiency, (i) vitamin B_12_ deficiency, (j) inflammation based on C-reactive protein and (k) inflammation based on alpha-1-acid glycoprotein in South Africa from 1997 to 2022, differentiated by age groups. Linear regressions were performed, and trend lines and equations were indicated for all studies (solid lines) and additionally for national representative data only (dash lines), where more than one national study was conducted. The slope depicts the change in percentage per year, and *R* ^2^ indicates the proportion of the variance in the dependent variable (% deficiency) that is predictable from the independent variable (time in years)

Vitamin A deficiency ranged from 1·4 % to 67·4 % during 1997–2022 (Table 2 and Fig. 2(a)), declining from 64 % in 2005 to 44 % in 2012 in the national surveys^(19,20)^ and about 2 % to 3 % per year (Fig. 2(a)). In regional studies prior to 2001, the prevalence of vitamin A deficiency ranged from 34·7 % to 67·3 %, except in a small study where it was 10 % in urban infants aged 1–6 months in the Western Cape^(27)^. Between 2008 and 2022, vitamin A deficiency ranged from 1·4 % to 16·1 %, except in a study from rural Limpopo, where prevalence was 57 % in children aged 2 years from 2009–2011^(28)^. Five studies measured vitamin A status of rural children aged 0–6 years in KwaZulu-Natal; vitamin A deficiency declined from above 40 % in the early 2000’s^(29–31)^ to below 10 %, as evident from data collected in two studies in 2010 and 2017^(18,32)^. Most studies used the cut-off of retinol < 20 μg/dl (< 0·7 μmol/l) to define vitamin A deficiency, which is in line with the WHO guidelines^(9)^. The concentration of vitamin A biomarkers was adjusted to account for the effects of inflammation in only one study in preschool children^(18)^.

**Table 2:** Vitamin A status of South-African infants, children and adolescents from 1997 to 2022

Province	Year of study	Age, years†	Sex	Ethnicity (race)	Sample size, *n*	Retinol (µg/dl)				VAD Serum retinol < 20 µg/dl (%)	VAD corrected for inflammation (%)	Reference
						Mean	sd	Mean	95 % CI			
0–6 years old, rural and urban												
National	2012	< 5	221M 217F	All	438			21·5	20·1, 22·6	43·6		Shisana et al., 2013^(20)^
National	2005	1–9	M F	All	1388			17·8	17·2, 18·4	63·6		Labadarios et al., 2007^(19)^
								18·4	17·8, 19·0‡			
0–6 years old, rural												
National	2005	1–9	M F	All	600			17·4	16·4, 18·3	67·3		Labadarios et al., 2007^(19)^
								17·8	16·8, 18·9§			
KZN	2017	2–4	22M 17F	NS	39					9·1M 0·0F		Makanjana and Naicker, 2020^(32)^
KZN	2017	4–5	27M 24F	NS	51					7·2M 8·3F		Makanjana and Naicker, 2020^(32)^
KZN	2011	< 5	M F	NS	140			26·0	25·0, 27·1	13·6	6·6**	Faber et al., 2015^(18)^
KZN	2000	6 m-1	99M 95F	NS	194	27·4	7·8			16·1††		Smuts et al., 2005^(29)^
KZN	1998	2–5	77M 87F	NS	164			–		50·0		Faber et al., 2001^(30)^
KZN	1998	6 m-2	50M 47F	Zulu (B)	97	22·1	6·5			39·2		Faber and Benade, 2000^(31)^
LP	2009–2011	2	M F	NS	314	20·2	6·2			57·0		MAL-ED, 2017^(28)^
LP	2011	< 5	M F	NS	206			28·8	27·8, 29·8	11·2	5·5**	Faber et al., 2015^(18)^
0–6 years old, urban												
National	2005	1–9	M F	All	688			18·1	17·4, 18·9	60·7		Labadarios et al., 2007^(19)^
								18·8	18·0, 19·6||			
FS	1998	< 1–5	171M 197F	NS	368	21·6	6·2			18·8		Dannhauser et al., 2000^(102)^
NC	2016	3–5	43M 52F	NS	95	32·1	9·5			6·7		van Stuijvenberg et al., 2019^(61)^
NC	2011	< 5	M F	NS	194			29·7	28·7, 30·8	9·8	2·2**	Faber et al., 2015^(18)^
NC	2008	1–6	119M 124F	NS	243			31·3	31·3, 32·3*	5·8		van Stuijvenberg et al., 2012^(103)^
WC	2011	< 5	M F	NS	207			29·8	28·8, 30·8*	8·2	3·1**	Faber et al., 2015^(18)^
WC	2000	1–6m	51M 62F	NS	113	26·9	7·2			10·0		Sibeko et al., 2004^(27)^
WC	1999–2000	6m	NS	NS (B)	46	30·5	7·4			–		Oelofse et al., 2002^(104)^
						28·8	6·6					
Primary school, rural												
FS‡‡	2008 and 2013	7–15	35M 38F	NS	73	35·1	8·5			1·4		Egal and Oldewage-Theron, 2017^(37)^
NW	2012	6–12	87M 80F	NS	167			–		4·8		van der Hoeven et al., 2016^(44)^
Primary school, urban												
NW	2010	6–11	213M 195F	NS	408	29·7	6·0¶			3·5¶		Taljaard et al., 2013b^(39)^

VAD, vitamin A deficiency, M, male; F, female; KZN, KwaZulu-Natal; NS, not specified; B, black; LP, Limpopo Province; FS, Free State; NC, Northern Cape; WC, Western Cape; NW, North West; CRP, C-reactive protein.

* Median (25th, 75th percentile).

† Age of participants reported in years, unless indicated differently in months (m).

‡ Only participants with CRP < 10 mg/l were included, *n* 1020.

§ Only participants with CRP < 10 mg/l were included, *n* 436.

|| Only participants with CRP < 10 mg/l were included, *n* 584.

¶ Participants were divided randomly into four groups.

** Corrected for inflammation according to Kongsbak et al., 2006.

†† Only participants with CRP < 12 mg/l were included.

‡‡ Ages up to 15 years included.

### Anaemia and iron status

Anaemia varied from 5·4 % to 75·0 % during 1997–2022 in SA (Table 3 and Fig. 2(b)). Among 0- to 6-year-olds, national data for anaemia declined from 27·9 % in 2005 to 10·7 % in 2012 but inclined again to 61 % in 2016^(19,20,25)^. Regional studies ranged from 21·7 % to 75·0 %, generally showing a decline over time. However, infants had a persistently high prevalence from 36·2 % to 54·0 %. Prevalence in regional studies ranged between 28 % and 50 % in rural^(32,33)^ and 33·9 % and 39·2 % in urban 0- to 6-year-olds^(34–36)^. Among primary school children, anaemia prevalence in rural areas ranged from 3·9 % to 56·9 % with all data collected from 2008 to 2013^(37,38)^, and in urban areas from 5·4 % to 19·2 %, with data collected from 2009 to 2019^(39,40)^. Authors generally used WHO guidelines for cut-offs^(41)^ but few mentioned corrections for altitude.

**Table 3:** Anaemia and Fe status of South-African infants, children and adolescents from 1997 to 2022^‘^

Province	Year of study	Age† years	Sex	Ethnicity (race)	Sample size*, n*	Hb (g/dl)		Ferritin (μg/l)		TfR (mg/l)		Anaemia (%) Hb < 11	Iron Deficiency (%) SF < 12	Iron Deficiency Anaemia (%)	Reference
						Mean	95 % CI	Mean	95 % CI	Mean	95 % CI				
0–6 years old, rural and urban															
National	2016	6m–5	M F	All	1137	–		–		–		61·0	–	–	NDoH, Stats SA, SAMRC, and ICF, 2019^(25)^
National	2012	< 5	249M 262F	All	511	12·2	12·0, 2·3	40·7	33·9, 47·6	–		10·7	8·1	1·9¶¶	Shisana et al., 2013^(20)^
National	2005	1–9	M F	All	1730	11·8	11·7, 11·8	38·0	35·9, 40·1	–		27·9	19·7||	7·6||||	Labadarios et al., 2007^(19)^
								37·3	35·3, 39·4§						
								33·4	31·0, 35·8||						
0–6 years old, rural															
National	2005	1–9	M F	All	706	11·8	11·7, 11·9	41·5	37·8, 45·1	–		24·6	16·2||	5·6||||	Labadarios et al., 2007^(19)^
								40·4	36·7, 44·0¶						
								36·2	32·7, 39·7||						
KZN	2017	2–4	23M 22F	NS	45	–		–		–		30·4M 50·0F	–		Makanjana and Naicker, 2020^(32)^
KZN	2017	4–5	28M 28F	NS	56	–		–		–		39·3M 28·6F	–		Makanjana and Naicker, 2020^(32)^
KZN	2002	6m–1	250M 229F	NS	479	–		–		–		49·7	–		Faber et al., 2007^(105)^
KZN	2000	6m–1	99M 95F	NS	194	Mean	sd	Mean	sd	–		40·2	18·3§§	–	Smuts et al., 2005^(29)^
						11·3	1·0								
KZN	1998	2–5	77M 87F	NS	164	10·6	1·3	17·7	14·2	–		54·0	33·0‡‡‡	–	Faber et al., 2001^(30)^
LP	2009–2011	2	M F	NS	314	11·0	1·1	31·2	31·2	–		42·0	–	–	MAL-ED, 2017^(28)^
LP	2012	3–5	136M 186F	NS	349	11·4	1·1	25·0	18·0	–		28·0	7·2	–	Motadi et al., 2015^(33)^
LP	1999–2000	1	M F	NS (B)	127	10·7	1·4	25·1	25·1	–		52·0	39·4	–	Mamabolo and Alberts, 2014^(42)^
LP	1999–2000	3	M F	NS (B)	143	11·9	1·2	20·8	14·1	–		21·7	32·9	–	Mamabolo and Alberts, 2014^(42)^
0–6 years old, urban															
National	2005	1–9	M F	All	1024	Mean	95 % CI	Mean	95 % CI	–		30·1	22·4||	8·9	Labadarios et al., 2007^(19)^
						11·7	11·6, 11·8	35·4	33·0, 37·9						
								35·1	32·7, 37·4**						
								31·2	27·9, 34·5||						
FS	1998	< 1–5	171M 197F	NS	368	–		–		–		50·5	18·1	–	Dannhauser et al., 2000^(102)^
GP	2011–2012	1	445M 449F	NS	894	–		14·9	14·0, 15·9	11·2	10·9, 11·5	–	41·9	–	Muriuki et al., 2020^(43)^
LP	2007 and 2008	< 5	32M 20F	NS	52	Mean	sd	–		–		75·0‡	–	–	Heckman et al., 2010^(106)^
						9·7	2·6								
NW	2016	1	87M 78F	NS	165	11·6·	1·7	–		–		33·9	–	–	Rikhotso et al., 2022^(34)^
NW††	2013–2015	6–9m	378M 363F	NS	750	Median	25th, 75th	Median	25th, 75th	–		36·5	16·0	10·4¶¶	Smuts et al., 2019^(35)^
						11·2	10·5, 12·1	24·9	16·0, 40·6						
						11·5	10·5, 12·3	25·3	16·4, 40·2						
						11·3	10·5, 12·1	25·4	15·3, 39·8						
WC	2013–2016	2w	47M 33F	NS	80	Mean	sd	Mean	sd	Mean	sd	–	–	–	Carter et al., 2021^(36)^
						15·4	2·7	218·8	136·6	–					
WC	2013–2016	6·5m	47M 33F	NS	80	10·9	0·9	26·1	17·8	–		49·2	23·1	13·0	Carter et al., 2021^(36)^
WC	2000	1–6m	51M 62F	NS	113	10·9	1·1	–		6·0·	1·8	50·0	–	–	Sibeko et al., 2004^(27)^
WC	1999–2000	6m	NS	NS (B)	46	10·8	1·0	–		–		–	–	–	Oelofse et al., 2002^(104)^
						10·3	1·0								
Primary school, rural															
FS‡‡	2008 and 2013	7–15	35M 38F	NS	73	13·2	1·3	–		–		3·9*	–	–	Egal and Oldewage-Theron, 2017^(37)^
KZN	2010	6–8	698M 688F	NS	1386	–		–		–		48·3*	–	–	Ajayi et al., 2017^(65)^
KZN	2010 & 2012	6–8	109M 72F	NS	181	–		–		–		56·9*	–	–	Gwetu et al., 2015^(38)^
KZN	2009	6–11	M F	NS	926	–		–		–		11·5*	7·3	–	Taljaard et al., 2013a^(107)^
NW	2012	6–12	87M 80F	NS	167	–		–		–		13·2*	17·4§§§	1·8***	van der Hoeven et al., 2016^(44)^
Primary school, urban															
EC	2019	6–12	664M 613F	NS	1277	12·3	0·9	–		–		19·2*			Beckmann et al., 2021^(40)^
NC	2009	5–11	111M 86F	NS	200	–		–		–		5·4*	3·3	–	Taljaard et al., 2013a^(107)^
NW	2010	6–11	265M 301F	NS (B)	566	Median	IQR	Median	IQR	Median	IQR	6·8*	13·9	5·6†††	Onabanjo et al., 2012^(45)^
						12·6	1·2 M	24·7	21·7 M	5·8	1·7 M				
						12·8	1·2 F	26·9	28·3 F	5·7	1·7 F				

IQR, interquartile range; TfR, transferrin receptor; SF, serum ferritin; M, male; F, female; KZN, KwaZulu-Natal; NS, not specified; LP, Limpopo Province; B, black; FS, free state; GP, Gauteng Province; NW, North West; WC, Western Cape; EC, Eastern Cape; NC, Northern Cape; CRP, C-reactive protein.

* Hb < 11.5 g/dl.

† Age of participants reported in years, unless indicated differently in months (m) or weeks (w).

‡ Anaemia defined as Hb falling > 2 sd below the mean of age-specific, altitude-adjusted Hb values.

§ Only participants with CRP < 10 mg/l were included, *n* 1116.

|| Only participants aged 1–5 years were included.

¶ Only participants with CRP < 10 mg/l were included, *n* 477.

** Only participants with CRP < 10 mg/l were included, *n* 639.

†† Participants were divided randomly into more than one group.

‡‡ Ages up to 15 years included.

§§ Ferritin of participants with CRP > 5 was adjusted with 0·65 as correction factor.

|||| Hb < 11 g/dl (0- to 4-year-olds) or Hb < 11·5 g/dl (5- to 11-year-olds) and ferritin < 12 µg/l.

¶¶ Hb < 11 g/dl and Fer < 12 µg/l.

*** Hb < 11·5 g/dl and Fer < 15 µg/l.

††† Hb < 11·5 g/dl and Fer < 12 µg/l.

‡‡‡ SF < 10 µg/l.

§§§ SF < 15 µg/l.

ID ranged from 3·3 % to 41·9 % (Table 3 and Fig. 1(c)), declining in younger children according to the national surveys from 19·7 % in 2005 to 8·1 % in 2012^(19,20)^. Among 0- to 6-year-olds, ID ranged from 7·2 % to 39·4 % in rural areas^(33,42)^ and 16 % to 41·9 % in urban areas^(35,36,43)^. In recent studies, ID in urban 0- to 6-year-olds varied from 16 % to 41·9 %^(35,36,43)^. In studies on primary school children conducted between 2009 and 2012, ID in rural areas was 7·3 % to 17·4 %^(39,44)^ and 3·3 % to 13·9 % in urban areas^(39,45)^. Most studies used WHO guidelines according to age using ferritin < 12 µg/l for infants and young children (0–23 months) and toddlers and preschoolers (24–59 months) and ferritin < 15 µg/l for children (5 to less than 10 years) and adolescents (10 to less than 20 years)^(46)^.

IDA prevalence was between 1·8 % and 13·0 % (Table 3 and Fig. 1(d)). According to the national surveys, IDA declined in 0- to 6-year-olds from 7·6 % to 1·9 % from 2005 to 2012^(19,20)^. Among 0- to 6-year-olds, there were regional data only for two studies from urban areas which collected data during 2013–2016 indicated that IDA prevalence was 10·4 %^(35)^ and 13·0 %^(36)^. Data in primary school children represented by one study each in rural and urban areas showed a prevalence of 1·8 % in 2012^(44)^ and 5·6 % in 2010^(45)^, respectively.

### Zinc status

Zn deficiency ranged between 8 % and 47·8 % among all age groups. In 0- to 6-year-olds, Zn deficiency was high in rural areas, ranging from 39·3 % to 47·8 % (Table 4 and Fig. 2(e)), with national and regional data agreeing but no urban data. In primary school children, Zn deficiency prevalence varied, ranging from 8 % to 46 % in urban areas and 25·0 % to 75·5 % in rural areas^(37,39,44)^. High prevalences of 25 % to 75 % persisted in the most recent data collected in 2012–2013^(33,37,44)^. All studies used cut-offs for Zn deficiency defined by the International Zinc Nutrition Consultative Group (IZiNCG).

**Table 4:** Zn status of South-African infants, children and adolescents from 1997 to 2022

Province	Year of study	Age, years†	Sex	Ethnicity (race)	Sample size, *n*	Zn (μg/dl)				Zn deficiency (%) (< 65 μg/dl)	Reference
						Mean	sd	Mean	95 % CI		
0–6 years old, rural and urban											
National	2005	1–9	M F	All	1730			68·7	66·5, 70·8	45·3	Labadarios et al., 2007^(19)^
0–6 years old, rural											
National	2005	1–9	M F	All	706			69·3	65·6, 73·0	39·3	Labadarios et al., 2007^(19)^
KZN	2000	6m–1	99M 95F	NS	194	73·9	15·0‡			46·8	Smuts et al., 2005^(29)^
LP	2012	3–5	136M 186F	NS	349	66·3	28·8			42·6	Motadi et al., 2015^(33)^
0–6 years old, urban											
National	2005	1–9	M F	All				68·4	65·7, 71·0	47·8	Labadarios et al., 2007^(19)^
WC	1999–2000	6m	NS	NS (B)	46	79·3	12·1§			–	Oelofse et al., 2002^(104)^
						69·1	15·8				
Primary school, rural											
FS||	2008 and 2013	7–15	35M 38F	NS	73	83·0	1·24			25·0	Egal and Oldewage-Theron, 2017^(37)^
NW	2012	6–12	87M 80F	NS	167			–		75·5	van der Hoeven et al., 2016^(44)^
Primary school, urban											
GP	2004	7–11	M F	NS (B)	133	66·4	21·5			46·0*	Samuel et al., 2010^(108)^
NW||	2010	6–11	213M 195F	NS	408	80·1	13·7			12·1	Taljaard et al., 2013b^(39)^

M, male; F, female; B, KZN, KwaZulu-Natal; NS, not specified; LP, Limpopo WC, Western Cape; B, black; FS, Free State; NW, North West; GP, Gauteng Province; CRP, C-reactive protein.

* Zn < 70 µg/dl.

† Age of participants reported in years, unless indicated differently in months (m).

‡ Participants with CRP > 12 mg/l were excluded.

§ Participants were divided randomly into more than one group.

|| Ages up to 15 years included.

### Urinary iodine concentration

The prevalence of urinary iodine concentrations below 100 ug/l in children was measured only in one national and three regional studies and ranged from 0 % to 68 % (Table 5 and Fig. 2(f)). The prevalence in 1- to 9-year-olds in both rural and urban areas of a national survey in 2005^(19)^ was between 0 % and 28·8 %, with excessive levels of urinary iodine in two areas (Free State and Northern Cape province). In an older study of primary or school children, 68·8 % iodine insufficiency was found in the most Northern rural district (Vhembe) of Limpopo, where only 18·2 % of the households were found to use salt that was adequately iodised^(47)^. Later (2012–2013), in rural and peri-urban Mopani district just to the south, the prevalence was 15·5 %^(48)^. The WHO defines iodine insufficiency in children under 2 years when the median urinary iodine concentration (UIC) of a population is < 100 μg/l while a median UIC of 50–99 μg/l, 20–49 μg/l and < 20 μg/l indicates mild, moderate and severe iodine deficiency, respectively^(49)^. Concentrations of 200–299 μg/l are regarded as above requirements and ≥ 300 μg/l as excessive^(50)^.

**Table 5:** Iodine status of South-African infants, children and adolescents from 1997 to 2022

Province	Study year	Age, years†	Sex	Ethnicity (race)	Sample size, *n*	Urinary iodine (μg/l)		Low urinary iodine (%) (< 100 μg/l)	Reference
						Median	25th, 75th		
0–6 years old, rural and urban									
National	2005	1–9	M F	All	1332	214·8	118·2, 367·4	26·8	Labadarios et al., 2007^(19)^
EC	2005	1–9	M F	All	205	204·2	92·9, 361·3	28·8	Labadarios et al., 2007^(19)^
FS	2005	1–9	M F	All	65	321·0	180·5, 512·7	10·8	Labadarios et al., 2007^(19)^
GP	2005	1–9	M F	All	325	192·6	114·4, 304·1	21·3	Labadarios et al., 2007^(19)^
KZN	2005	1–9	M F	All	223	263·0	160·3, 430·4	11·7	Labadarios et al., 2007^(19)^
MP	2005	1–9	M F	All	148	180·5	110·4, 298·5	20·3	Labadarios et al., 2007^(19)^
NC	2005	1–9	M F	All	20	777·7	507·4, 836·6	0·0	Labadarios et al., 2007^(19)^
LP	2005	1–9	M F	All	146	210·2	127·7, 361·3	15·8	Labadarios et al., 2007^(19)^
NW	2005	1–9	M F	All	87	161·2	91·9, 309·5	25·2	Labadarios et al., 2007^(19)^
WC	2005	1–9	M F	All	113	213·0	126·7, 389·4	17·7	Labadarios et al., 2007^(19)^
0–6 years old, rural									
National	2005	1–9	M F	All	706	197·5	107·8, 349·0	–	Labadarios et al., 2007^(19)^
0–6 years old, urban									
National	2005	1–9	M F	All	1024	230·3	131·4, 376·4	–	Labadarios et al., 2007^(19)^
NW	2013	2–4m	M F	Tswana(B)	92	373	202, 627	4·0	Osei et al., 2016^(83)^
Primary school, rural									
LP	2012–2013	6–12	M F	NS	116	386	200, 525	15·5	Mabasa et al., 2018^(48)^
LP	2003	6–14	M F	NS	664	Mean	sd	68·8	Mabapa et al., 2014^(47)^
						82	1002		

M, male; F, female B, black; EC, Eastern Cape; FS, Free State; GP, Gauteng Province; KZN, KwaZulu-Natal; MP, Mpumalanga Province; NC, Northern Cape; LP, Limpopo Province; NW, North West; WC, Western Cape; B, black; NS, not specified.

† Age of participants reported in years, unless indicated differently in months (m).

### Vitamin D status

Vitamin D status was assessed in regional studies in urban children only, including new-borns^(51)^, two samples in separate settings of 6- to 10-week-old infants^(52)^ and primary school children in the Bone Health sub-cohort of the Birth-to-Twenty cohort (Table 6 and Fig. 2(g))^(26,53)^. Thirty-three percent of new-borns were deficient^(51)^, and in the two settings of 6- to 10-week-old infants, 76·4 % and 86·5 % infants were deficient, whereas 12·1 % and 22·4 % were insufficient^(52)^. At the age of 10 years, 7 % of the children were vitamin D deficient and 19 % insufficient, whereas 5 % were deficient and 35 % insufficient when combining the measurements over all the years from 11 to 20 years old (*n* 423)^(26)^. A serum 25-hydroxyvitamin D [25(OH)D] concentration of below 20 ng/ml (50 nmol/l) is considered to be vitamin D-deficient and a 25(OH)D of 21–29 ng/ml (52·5–72·5 nmol/l) to be insufficient^(54)^.

**Table 6:** Vitamin D status of South-African infants, children and adolescents from 1997 to 2022

Province	Year of study	Age†, years	Sex	Ethnicity (race)	Sample size, *n*	25(OH)D (nmol/l)		Vit D deficiency (%) (< 50 nmol/l)	Vit D insufficiency (%) (50–74 nmol/l)	Reference
						Mean	sd			
0–6 years old, urban										
GP	2013–2014	1d	M F	NS (B)	291	41·9	21·0	33·0	–	Velaphi et al., 2019^(51)^
WC	2012–2015	6–10w	M F	NS	411	41·2	15·3	76·4	22·4	Ncayiyana et al., 2021^(52)^
WC	2012–2015	6–10w	M F	NS	363	31·0	17·2	86·5	12·1	Ncayiyana et al., 2021^(52)^
Primary school, urban*										
GP	2001–2010	11	M F	NS (B,W)	99	58·6	5·8	5·0	35·0	Poopedi et al., 2015^(26)^
GP	2001–2010	13	M F	NS (B,W)	82	58·6	7·7			Poopedi et al., 2015^(26)^
GP	2000	10	198M 187F	NS (B,W)	385	M: B 100 ± 34; W 129 ± 37 F: B 86 ± 31; W 112 ± 35		7·0	19·0	Poopedi et al., 2011^(53)^
Adolescents, urban*										
GP	2001–2010	15	M F	NS (B,W)	76	55·6	7·7			Poopedi et al., 2015^(26)^
GP	2001–2010	17	M F	NS (B,W)	90	60·6	7·7			Poopedi et al., 2015^(26)^

GP, Gauteng Province; M, male; F, female; B, black; WC, Western Cape; NS, not specified; W, white.

* The same sample of children longitudinally assessed.

† Age of participants reported in years, unless indicated differently in weeks (w), or days (d).

### Folate and vitamin B_12_ status

Folate and vitamin B_12_ status was only measured in 0- to 6-year-olds. Folate deficiency ranged between 0·1 % and 22·8 % and vitamin B_12_ deficiency between 0 % and 20·9 % (Table 7 and Fig. 2(h) and (i)). National and regional data collected in 2005 and after reported folate deficiency below 0·4 %^(19,36)^. Data collected during 2013–2016 showed vitamin B_12_ deficiency of 20·9 %^(36)^. Folate deficiency in all age groups was defined as serum/plasma folate < 4 ng/ml (< 10 nmol/l) or red blood cell folate < 151 ng/ml (< 340 nmol/l) in line with WHO cut-off points when using homocysteine concentrations as metabolic indicator (WHO, 2015). Various cut-offs were used for vitamin B_12_ deficiency, including plasma vitamin B_12_ < 107 pmol/l (< 145 pg/ml) and 155·7 pmol/l (210·7 pg/ml)^(42,55)^.

**Table 7:** Folate and vitamin B_12_ status of South-African infants and children from 1997 to 2022

Province	Year of study	Age*, years	Sex	Ethnicity (race)	Sample size *n*	RBC and serum folate (nmol/l)		Folate deficiency (%) (serum < 4 ng/ml) (RBC < 151 ng/ml)	Vit B_12_ (pg/ml)		Vit B_12_ deficiency (%) (< 145 pg/ml)	Reference
						Mean	95 % CI					
0–6 years old, rural and urban												
National	2005	1–9	M F	All	1502	1397	1338, 1456†	0·2†	–		–	Labadarios et al., 2007^(19)^
						39·1	38·0, 40·1‡					
0–6 years old, rural												
National	2005	1–9	M F	All	576	1263	1199, 1327†	0·4†	–		–	Labadarios et al., 2007^(19)^
						38·8	37·0, 40·5‡					
LP	1999–2000	1	M F	NS (B)	127	Mean	sd	22·8‡	Mean	sd	10·2	Mamabolo and Alberts, 2014^(42)^
						8·1	4·0‡		362	219		
LP	1999–2000	3	M F	NS (B)	143	6·7	2·1‡	19·6†	449	206	0·0	Mamabolo and Alberts, 2014^(42)^
0–6 years old, urban												
National	2005	1–9	M F	All	926	Mean	95 % CI	0·1†	–		–	Labadarios et al., 2007^(19)^
						1481	1393, 1569†					
						39·3	38·0, 40·5‡					
WC	2013–2016	6·5m	47M 33F	NS	80	Mean	sd	0·1†	Mean	sd	20·9^#^	Carter et al., 2021^(36)^
						1755	685†		284	168		

RBC, red blood cell; Vit, vitamin; M, male; F, female; LP, Limpopo Province; NS, not specified; B, black; WC, Western Cape.

* Age of participants reported in years, unless indicated differently in months (m).

# Vit B12 < 210 pg/ml.

† Red blood cell.

‡ Serum.

### Inflammation

The prevalence of elevated CRP and AGP ranged from 4·8 % to 26·1 % and 10·4 % to 41·8 %, respectively (Table 8 and Fig. 2(j) and (k)). When considering CRP, low-grade inflammation was around 8 % in the 2005 national data and ranged between 15 % and 26 % in regional studies when the cut-off of 5 mg/l currently suggested by the WHO^(56)^ was used^(18,35,39,43)^, except in one study in primary school farm children from a rural area^(44)^, where it was 4·8 %. In the studies that defined low-grade inflammation as CRP > 10 mg/l, the prevalence of low-grade inflammation was generally below 10 %, and there were no clear trends among different ages, years of study or urban and rural areas. The prevalence of AGP > 1 g/l, particularly useful for monitoring the later stages of inflammation, ranged from 10·4 % to 41·8 % in rural and urban infants and children aged 0–6 years, with no apparent difference between areas and no data for primary school children^(18,29,35)^.

**Table 8:** Inflammatory status of South-African infants, children and adolescents from 1997 to 2022

Province	Year of study	Age, years†	Sex	Ethnicity (race)	Sample size, *n*	CRP (mg/l)		Elevated CRP (> 5mg/l) (%)	Elevated AGP (> 1g/l) (%)	Reference
						Mean	95 % CI			
0–6 years old, rural and urban										
National	2005	1–9	M F	All	1422	3·2	2·7, 3·7	6·9||	–	Labadarios et al., 2007^(19)^
0–6 years old, rural										
National	2005	1–9	M F	All	597	3·5	2·8, 4·2	7·7||	–	Labadarios et al., 2007^(19)^
KZN	2011	< 5	M F	NS	140	–		15·4	18·4	Faber et al., 2015^(18)^
KZN	2000	6 m–1	99M 95F	NS	194	–		12·4¶	41·8	Smuts et al., 2005^(29)^
LP	2011	< 5	M F	NS	206	–		16·1	17·6	Faber et al., 2015^(18)^
0–6 years old, urban										
National	2005	1–9	M F	All	825	3·0	2·3, 3·7	6·3||	–	Labadarios et al., 2007^(19)^
GP	2011–2012	1	445M 449F	NS	894	–		17·6		Muriuki et al., 2020)^(43)^
NC	2011	< 5	M F	NS	194	–		16·4	10·4	Faber et al., 2015^(18)^
NC	2008	1–6	119M 124F	NS	243	–		6·4||	–	van Stuijvenberg et al., 2012^(103)^
NW	2013–2015	6–9 m	378M 363F	NS	750	–		15·2	31·5	Smuts et al., 2019^(35)^
WC	2013–2016	6·5 m	47M 33F	NS	80	Mean	sd	–	–	Carter et al., 2021^(36)^
						4·3	6·0			
WC	2011	< 5	M F	NS	207	–		21·1	11·9	Faber et al., 2015^(18)^
Primary school, rural										
EC‡	2013–2014	6–18	116M 118F	NS	234	2·3*		19·0**	–	Oldewage-Theron et al., 2017^(68)^
NW	2012	6–12	87M 80F	NS	167	–		4·8	–	van der Hoeven et al., 2016^(44)^
Primary school, urban										
NW	2010	6–11	213M 195F	NS	408	–		26·1	–	Taljaard et al., 2013b^(39)^
NW	2010	6–11	265M 301F	NS (B)	566	3·7 (3·1) M^§^		–	–	Onabanjo et al., 2012^(45)^
						3·3 (2·7) F^§^				

CRP, C-reactive protein; AGP, alpha-1 acid glycoprotein; IQR, interquartile range; M, male; F, female; KZN, KwaZulu-Natal; NS, not specified; LP, Limpopo Province; GP, Gauteng Province; NC, Northern Cape; NW, North West; WC, Western Cape; EC, Eastern Cape; B, black.

* Median

† Age of participants reported in years, unless indicated differently in months (m).

‡ Participants up to 18 years included.

§ Median (IQR)

|| CRP > 10 mg/l

¶ CRP > 12 mg/l

** CRP > 3 mg/l

## Discussion

This systematic review provides a comprehensive overview of the biochemical nutritional status of South African infants, children and adolescents from 1997 to 2022. This review included national surveys and regional studies. Micronutrient deficiencies varied widely depending on age, year of data collection, geographical area and rural or urban setting, as well as socio-economic status. Anaemia and ID ranged from 5·4 % to 75·0 % and 3·3 % to 41·9 %, respectively, while vitamin A deficiency ranged from 1·4 % to 67·4 % and Zn deficiency from 8·0 % to 75·5 %.

Vitamin A deficiency is associated with substantial morbidity and mortality from common childhood infections and is the most important preventable cause of childhood blindness^(9)^. Although data is sparce, this systematic review indicates a possible improvement in vitamin A deficiency in SA. This is demonstrated when viewing data over time for a specific area or province. For example, vitamin A deficiency declined from above 40 % in the early 2000’s to below 10 % after 2020 in rural 0 to 6-year-old children from KwaZulu-Natal^(18,29–32)^. The improvement could likely be attributed to the NFFP and routine periodic high-dose VAS at primary health care level in children under five, implemented in 2003 and 2002, respectively^(11,12)^. Coverage of VAS in 6- to 59-month-old children in SA has increased from 25 % in 2006/07 to 57 % in 2015/16,^(57)^ and fortified staple foods were shown to provide more than 50 % of total vitamin A intake in rural children^(18)^. Whether vitamin A deficiency has improved in SA will need to be confirmed by a national survey. Although the national and regional studies indicate a similar downward trend, the regional study results generally show a 20 % lower prevalence than national studies. The prevalence of vitamin A deficiency may be overestimated when not corrected for inflammation, as advised by the WHO^(56,58)^. However, some research also suggests that it could be underestimated^(59,60)^. Only one study adjusted for inflammation, showing that vitamin A deficiency decreased to less than half the original estimated prevalence when adjustments were made^(18)^. Careful consideration of blanket vitamin A supplementation is warranted in areas with low prevalence of vitamin A deficiency. For instance, children with regular consumption of organ meat have been identified to be at risk of hypervitaminosis^(61)^. Therefore, it is recommended that the vitamin A status of children be assessed at national level at least every 10 years^(62)^.

Anaemia was most prevalent in 0- to 6-year-old infants and children from both urban (4·8 % to 75 %) and rural (21·7 % to 54·0 %) areas, with a persistently high prevalence in infants (33·9 % to 52·0 %). This finding agrees with others, who reported a prevalence of 52·0 % in 1-year-olds in a review of South African children under five during 1997–2021^(17)^ and the risk being highest for anaemia in 6- to 24-month-olds from Namibia^(63)^. Regional data collected after 2013 in SA in 0- to 6-year-olds still generally indicated a high anaemia prevalence between 28 % and 50 %, though this is lower than the WHO-reported prevalence of 60 % in Africa during 2019 (aged 6–59 months). These regional findings agreed with the decrease in global anaemia from 48·0 % in 2000 to 39·8 % in 2010 in this age group^(64)^. Authors of the South African Demographic and Health survey of 2016 cautioned that the striking 61 % prevalence of anaemia in children aged 0–59 months should be interpreted cautiously. However, this alarming prevalence warrants further investigation^(25)^. In rural primary school-age children from low socio-economic areas with high levels of poverty, food insecurity and high numbers of infectious diseases, anaemia was above 40 %^(38,65)^. Interestingly, anaemia was reported at 13 % and less in similar severely impoverished areas in two studies. One study with lower prevalence reported that 91·3 % of households had a vegetable garden,^(37)^ and the other was conducted at a farm school, reporting cereal, cereal products and meat, especially fortified maize meal porridge and bread, as the main sources of Fe and other micronutrients^(44)^. It is important to note that not all studies adjusted for altitude and some used capillary blood instead of venous blood^(66)^.

ID in children under five declined from about 20 % in 2005 to 10 % in 2012 according to South African national surveys^(19,20)^. Results from regional studies showed a similar declining trend. However, higher prevalences of up to 39·4 % in 0- to 6-year-olds and 17·4 % in primary school children were reported in some regional studies from rural areas^(42,44)^. Higher prevalences of 23·1 % and 41·9 % were also reported after 2010 in urban 0- to 6-year-olds from low socio-economic status^(36,43)^. The South African national prevalence of 10 % for ID in 2012 is lower compared to the ID prevalence of ∼18 % found by a global review covering all studies ever performed in children under five until March 2021^(67)^. However, the ID prevalence found in most of the regional studies reviewed here was similar to the 34 % (52 % when adjusted for inflammation) found by a study of ID in African 0- to 8-year-old children from Kenya, Uganda, Burkina Faso, SA, and The Gambia in 2011–2012^(43)^. The lack of reporting of TfR values and categorisation into ID erythropoiesis limited the interpretation of Fe status, particularly due to the high prevalence of low-grade inflammation in South African children^(18,35,68)^. Ferritin is an acute-phase protein which increases when inflammation is present, possibly leading to the underestimation of ID^(9)^. Several strategies can be used to correct for the influence of inflammation but not all the included studies adjusted for inflammation. Serum TfR levels reflect the intensity of erythropoiesis and, therefore, the demand for Fe^(69)^. Transferrin receptor is also less influenced by inflammation, making it a more reliable Fe status indicator in settings with a high prevalence of low-grade inflammation^(69)^.

IDA was only reported in one national survey in 2005 at around 8 %^(19)^. Overall, the prevalence of IDA was less than 14 % in regional studies and generally about half or less than half of the percentage of ID and anaemia in each study. This may indicate that, firstly, ID was not severe enough to cause anaemia in all cases and, secondly, that other factors such as micronutrient deficiencies or parasite infections also contributed to anaemia^(70–72)^. Only one study in urban primary school children from North West province showed that ID may have been the major contributor to anaemia, as IDA and anaemia prevalence was similar at about 6 %^(45)^. ID and other micronutrient deficiencies (vitamin A, vitamin B_12_ and folate) remain important causes of anaemia leading to serious developmental deficits in children like impairing brain and immune function^(41)^.

In children, insufficient plasma Zn levels could reduce appetite, slow down growth, impair immune function^(73)^, and if persist, could lead to wasting^(74)^. Although data are sparse with the most recent data collected in 2014, the prevalence of Zn deficiency appears to be consistently high in South African 0- to 6-year-olds since 1999, ranging from 39·3 % to 47·8 %. National data from 2005 and the few regional studies agree. Most low-and middle-income countries have a high prevalence of Zn deficiency among children (> 20 %), with the prevalence being similar or even higher than SA in other African countries^(75,76)^. This high prevalence is mostly attributed to the lack of absorbable Zn in the diet^(76)^ and persists in South African children despite the NFFP of maize meal and wheat flour since 2003, improving the nutrient adequacy ratio of Zn in the diet to above 100 % in 1- to 8-year-old children^(77)^. The persistent poor Zn status is likely partially attributable to the high phytic acid content of plant-based diets, which inhibit Zn absorption^(76,77)^. Despite being the best indicator of Zn status, plasma/serum Zn concentration is sensitive to inflammation, fasting or eating and diurnal rhythm^(78,79)^.

The global effort to eradicate iodine deficiency has been largely successful^(80)^ and reflects in South African data. In SA, iodisation of table salt of 35–65 mg iodine/kg salt is mandatory^(81)^. Therefore, iodine deficiency in SA is likely to be low, but certain rural areas may lag behind^(48)^. The impact of mandatory salt reduction strategies in certain processed foods in SA and the possible impact on iodine intake is still uncertain^(82)^. Furthermore, since urinary iodine concentrations were found to be excessive in some areas in the only national survey conducted among children and infants in 2005^(83)^, and considering the potential effect of the mandatory salt reduction policy on iodine intake,^(82)^ the recommendation to collect national data on iodine status every 5 years will be prudent to follow^(84,85)^.

Vitamin D deficiency was low (5 %) among the small sample of urban 10- to 17-year-olds^(26,53)^ but moderate to very high at 33 % to 87 % in urban infants^(51,52)^. Apart from its role in bone health, vitamin D plays an important role in infectious disease and inflammation^(86)^. Despite abundant sunshine, a vitamin D deficiency prevalence of 25 % was recently estimated by a meta-analysis across Africa^(87)^. It is suggested that Black Africans may be at higher risk for vitamin D deficiency due to their dark skin^(53,88)^. The high vitamin D deficiency in South African infants is alarming and concurs with other studies in Africa^(87)^. Breast-fed infants may be at higher risk for vitamin D deficiency if the mother has low vitamin D levels and the infant does not receive supplemental vitamin D^(51)^.

Folate and vitamin B_12_ deficiencies are the most common causes of macrocytic anaemia^(89)^. The very low prevalence of folate deficiency (< 1 %) in children since 2005 indicates the success of the fortification of maize meal and bread flour with folic acid (synthetic form of vitamin B_9_)^(19,36)^. However, vitamin B_12_ is not included in the fortification programme in SA, hence the 20 % deficiency in more recent data, but this evidence is from one small study only^(36)^. Good monitoring of vitamin B_12_ is important because high folic acid intake may mask vitamin B_12_ deficiency^(90)^. The high levels of folate should be monitored, even though 1000 µg/d folic acid for the general population is not associated with any adverse health outcomes^(90)^, some harmful consequences have been suggested due to the build-up of unmetabolised folic acid^(91)^.

The rising prevalence of inflammation, as indicated by elevated CRP levels, aligns with the findings of other research involving preschool children in Sub-Saharan African^(71,92)^, generally associated with recurrent acute and/or chronic infections (e.g. HIV, malaria and schistosomiasis), stunting and poor sanitation and/or poor drinking water quality^(71,92,93)^. Elevated CRP and AGP levels indicate the presence of inflammation and are used to interpret biomarkers of micronutrient status sensitive to inflammation, notably vitamin A, Fe and Zn^(56,92)^. They are also considered markers of environmental enteric dysfunction^(94)^. Environmental enteric dysfunction may be indicative of malabsorption, is associated with poor growth, and therefore important to consider when assessing nutritional status^(94)^.

Results showed high rates of anaemia, Fe and Zn deficiencies, especially among rural 0- to 6-year-olds, despite mandatory fortification of two staple foods (maize meal and bread) with, amongst others, Fe and Zn. The potential of fortified staple foods to improve micronutrient deficiencies in 6- to 23-months-olds is low because of their very high nutrient requirements and small amount of food consumed. Although increasing with age, the potential remains low in 2- to 5-year-olds^(95)^. Also, the bioavailability of particularly Fe and Zn is affected by the type of fortificant and the fortification vehicle^(96)^, and large variations in micronutrient content of fortified maize meal and bread have been reported^(97)^.

Consumption of fortified staple foods varies across geographical locations and between population groups in SA. Steyn *et al*.^(98)^ reviewed dietary intake studies in children aged 6–15 years and reported large variation not only in the percentage of children consuming fortified maize meal or bread but also in the amount eaten per day. It therefore follows that the contribution of fortified staples to total nutrient intake will vary across geographical locations and between population groups. For example, in children age 1–6 years, the contribution of fortified maize meal and/or bread to total intake for vitamin A has been reported to be more than 50 % in rural children, and approximately one-third in urban children^(18)^ while for 12-month-old consumers of maize meal and/or bread, less than 20 % of total vitamin A intake was from the fortified staples^(99)^.

Fortification of staple foods should not be seen as a stand-alone intervention and should rather be accompanied by nutrition education and complementary support to promote a diverse diet consisting of healthy and minimally processed foods. Although the NFFP and VAS are aimed specifically at improving micronutrient deficiencies, optimal implementation^(14)^ and improving the school food environment^(100,101)^ can contribute to children eating more diverse and healthier foods.

The observations on the biochemical nutritional status reported in this review are generally limited by the inherent characteristics of biomarkers, different methodologies and cut-offs used, as well as by the varying ages and age ranges in studies, making comparisons between studies difficult. Regional studies were generally not representative using non-probability sampling, and data were not weighted, leading to sparse data coverage and a risk for bias. In addition, data on adolescents are very limited.

In conclusion, vitamin A, folate and iodine status in infants, children and adolescents likely improved since 1997 in SA, possibly due to the vitamin A supplementation programme in children under five, iodisation of salt and NFFP of maize meal and wheat flour. Some excessive levels of iodine and folate were observed. However, anaemia, Fe and Zn deficiencies are still high, especially among rural 0- to 6-year-olds from low socio-economic status, with the deficiencies being worst in infants. More frequent national data are needed to confirm these findings based on older national surveys and unrepresentative regional studies and monitor the prevalence of micronutrient deficiencies as well as detect possible excessive levels among South African infants, children and adolescents.

## Supporting information

Malan et al. supplementary material Malan et al. supplementary material

## Supplementary material

For supplementary material accompanying this paper visit https://doi.org/10.1017/S136898002400137X
