# Supplementary material for: Biochemical assessment of the nutritional status of infants, children and adolescents in South Africa (1997–2022): a systematic review
Source: Public Health Nutr. 2024 Oct 21;27(1):e210. doi: 10.1017/S136898002400137X (PMC11604332; doi:10.1017/S136898002400137X)
Supplement: Malan et al. supplementary material [file S136898002400137Xsup001.docx]

**Biochemical assessment of the nutritional status of** **infants, children and adolescents in South Africa (1997-2022): A systematic review**

**Supplementary table 1: Joanna Briggs Institute (JBI) critical appraisal checklist for studies reporting prevalence data**

|  | **Article** | **Quality criteria***  **y=yes, n=no** | | | | | | | | |
| --- | --- | --- | --- | --- | --- | --- | --- | --- | --- | --- |
|  |  | **1** | **2** | **3** | **4** | **5** | **6** | **7** | **8** | **9** |
| 1 | Faber et al., 2015^(18)^ | y | y | y | y | y | y | y | y | y |
| 2 | Labadarios et al., 2007^(19)^ | y | y | y | y | y | y | y | y | y |
| 3 | Shisana et al., 2013^(20)^ | y | y | y | y | y | y | y | y | y |
| 4 | NDoH, Stats SA, SAMRC, and ICF, 2019^(25)^ | y | y | y | y | y | y | y | y | y |
| 5 | Poopedi et al., 2015^(26)^ | y | y | y | y | y | y | y | y | y |
| 6 | Sibeko et al., 2004^(27)^ | n | n | y | y | y | y | y | y | y |
| 7 | MAL-ED, 2017^(28)^ | n | n | y | y | y | y | y | y | y |
| 8 | Faber and Benade, 2000^(29)^ | y | y | y | y | y | y | y | y | y |
| 9 | Faber et al., 2001^(30)^ | y | y | y | y | y | y | y | y | y |
| 10 | Smuts et al., 2005^(31)^ | n | n | y | y | y | y | y | y | y |
| 11 | Makanjana and Naicker, 2020^(32)^ | n | n | y | y | y | y | y | y | y |
| 12 | Motadi et al., 2015^(33)^ | y | y | y | y | y | y | y | y | y |
| 13 | Carter et al., 2021^(34)^ | n | n | y | y | y | y | y | y | y |
| 14 | Rikhotso et al., 2022^(35)^ | n | n | y | y | y | y | y | y | y |
| 15 | Smuts et al., 2019^(36)^ | n | n | y | y | y | y | y | y | y |
| 16 | Egal and Oldewage-Theron, 2017^(37)^ | n | n | y | y | y | y | y | y | y |
| 17 | Gwetu et al., 2015^(38)^ | n | n | y | y | y | y | y | y | y |
| 18 | Beckmann et al., 2021^(39)^ | n | n | y | y | y | y | y | y | y |
| 19 | Taljaard et al., 2013b^(40)^ | n | n | y | y | y | y | y | y | y |
| 20 | Mamabolo and Alberts, 2014^(42)^ | y | y | y | y | y | y | y | y | y |
| 21 | Muriuki et al., 2020^(43)^ | n | n | y | y | y | y | y | y | y |
| 22 | Van der Hoeven et al., 2016^(44)^ | n | n | y | y | y | y | y | y | y |
| 23 | Onabanjo et al., 2012^(45)^ | n | n | y | y | y | y | y | y | y |
| 24 | Mabapa et al., 2014^(47)^ | y | y | y | y | y | y | y | y | y |
| 25 | Mabasa et al., 2018^(48)^ | y | y | y | y | y | y | y | y | y |
| 26 | Velaphi et al., 2019^(51)^ | n | n | y | y | y | y | y | y | y |
| 27 | Ncayiyana et al., 2021^(52)^ | n | y | y | y | y | y | y | y | y |
| 28 | Poopedi et al., 2011^(53)^ | y | y | y | y | y | y | y | y | y |
| 29 | van Stuijvenberg et al., 2019^(61)^ | n | n | y | y | y | y | y | y | y |
| 30 | Ajayi et al., 2017^(65)^ | y | n | y | y | y | y | y | y | y |
| 31 | Oldewage-Theron et al., 2017^(68)^ | y | y | y | y | y | y | y | y | y |
| 32 | Osei et al., 2016^(83)^ | n | n | y | y | y | y | y | y | y |
| 33 | Dannhauser et al., 2000^(103)^ | y | y | y | y | y | y | y | y | y |
| 34 | van Stuijvenberg et al., 2012^(104)^ | n | n | y | y | y | y | y | y | y |
| 35 | Oelofse et al., 2002^(105)^ | n | y | y | y | y | y | y | y | y |
| 36 | Faber et al., 2007^(106)^ | n | n | y | y | y | y | y | y | y |
| 37 | Heckman et al., 2010^(107)^ | n | n | y | y | y | y | y | y | y |
| 38 | Taljaard et al., 2013a^(108)^ | n | n | y | y | y | y | y | y | y |
| 39 | Samuel et al., 2010^(109)^ | n | n | y | y | y | y | y | y | y |

***Quality criteria**

1. Was the sample frame appropriate to address the target population?

2. Were study participants recruited in an appropriate way?

3. Was the sample size adequate?

4. Were the study subjects and setting described in detail?

5. Is the data analysis conducted with sufficient coverage of the identified sample?

6. Were valid methods used for the identification of the condition?

7. Was the condition measured in a standard, reliable way for all participants?

8. Was there appropriate statistical analysis?

9. Was the response rate adequate, and if not, was the low response rate managed appropriately?

**References (as presented in the main paper)**

1. Black RE, Victora CG, Walker SP *et al.* (2013) Maternal and child undernutrition and overweight in low-income and middle-income countries. *Lancet (London, England)* 382, 427-451.

2. Victora CG, Adair L, Fall C *et al.* (2008) Maternal and child undernutrition: consequences for adult health and human capital. *Lancet (London, England)* 371, 340-357.

3. Faber M, Malan L, Kruger HS *et al.* (2022) Potential of Egg as Complementary Food to Improve Nutrient Intake and Dietary Diversity. *Nutrients* 14, 3396.

4. Sayed N & Schönfeldt HC (2020) A review of complementary feeding practices in South Africa. *S Afr J Clin Nutr* 33, 36-43.

5. Bailey RL, West KP, Jr. Black RE (2015) The epidemiology of global micronutrient deficiencies. *Ann Nutr Metab* 66 Suppl 2, 22-33.

6. Barker D, Osmond C, Golding J *et al.* (1989) Growth in utero, blood pressure in childhood and adult life, and mortality from cardiovascular disease. *Br Med J* 298, 564-567.

7. Dewey KG & Begum K (2011) Long-term consequences of stunting in early life. *Matern Child Nutr* 7 Suppl 3, 5-18.

8. Miller JL (2013) Iron deficiency anemia: a common and curable disease. *Cold Spring Harb Perspect Med* 3, a011866.

9. World Health Organization (2011) *Serum retinol concentrations for determining the prevalence of vitamin A deficiency in populations*. Geneva: World Health Organization.

10. World Health Organization (2017) *C-reactive protein concentrations as a marker of inflammation or infection for interpreting biomarkers of micronutrient status*. Geneva: World Health Organization.

11. World Health Organization (1997) Vitamin A supplements : a guide to their use in the treatment of vitamin A deficiency and xerophthalmia / prepared by a WHO/UNICEF/IVACG task force, 2nd ed ed. Geneva: World Health Organization.

12. Department of Health (2004) Guidelines for the Implementation of Vitamin A Supplementation. Pretoria, South Africa. <https://pdf4pro.com/cdn/national-vitamin-a-supplementation-ceab8.pdf> (Acessed 12/05/2023).

13. Slemming W & Bamford L (2018) The new Road to Health Booklet demands a paradigm shift. *S Afr J Child Health* 12, 86-87.

14. Department of Basic Education (2016) Report on the Implementation Evaluation of the National School Nutrition Programme. Pretoria, South Africa. <https://www.dpme.gov.za/news/Documents/NSNP%20Report%20Final%2017092016.pdf>

(Acessed 12/05/2023).

15. Department of Health (2012) Integrated School Health Policy. Pretoria, South Africa. <https://core.ac.uk/download/pdf/141500394.pdf> (Acessed 12/05/2023).

16. Department of Health (2003) Regulations relating to the fortification of certain foodstuffs.

Pretoria, South Africa: Foodstuffs, Cosmetics and Disinfectants Act, No. R 2003. (Act No. 54 of 1972).

<https://www.agbizgrain.co.za/content/open/foodstuffs-cosmetics-and-disinfectants-act-no-54-of-1972> (Acessed 12/05/2023).

17. Turawa E, Awotiwon O, Dhansay MA *et al.* (2021) Prevalence of Anaemia, Iron Deficiency, and Iron Deficiency Anaemia in Women of Reproductive Age and Children under 5 Years of Age in South Africa (1997-2021): A Systematic Review. *Int J Environ Res Public Health* 18, 12799.

18. Faber M, van Jaarsveld PJ, Kunneke E *et al.* (2015) Vitamin A and anthropometric status of South African preschool children from four areas with known distinct eating patterns. *Nutr* 31, 64-71.

19. Labadarios D, Swart R, Maunder EMW *et al.* (2007) National Food Consumption SurveyFortifi cation Baseline (NFCS-FB) South Africa, 2005. Stellenbosch: Department of Health, UNICEF, GAIN.

20. Shisana O, Labadarios D, Rehle T *et al.* *South African National Health and Nutrition Examination Survey (SANHANES-1)*. Cape Town: Human Sciences Research Council.

21. Vorster HH, Oosthuizen W, Jerling JC *et al.* (1997) *The nutritional status of South Africans. A review of the literature from 1975 - 1996.* Durban, South Africa: Health Systems Trust.

22. Munn Z, Moola S, Riitano D *et al.* (2014) The development of a critical appraisal tool for use in systematic reviews addressing questions of prevalence. *Int J Health Policy Manag* 3, 123-128.

23. Viera AJ & Garrett JM (2005) Understanding Interobserver Agreement: The Kappa Statistic. *Fam Med* 37, 360-363.

24. Koo TK & Li MY (2016) A Guideline of Selecting and Reporting Intraclass Correlation Coefficients for Reliability Research. *J Chiropr Med* 15, 155-163.

25. National Department of Health - NDoH, Statistics South Africa - Stats SA, South African Medical Research Council - SAMRC, and ICF (2019) South African Demographic and Health Survey 2016: Key Indicators. Pretoria, South Africa: Rockville, MD, USA, 2016. NDoH, Stats SA, SAMRC, and ICF.

26. Poopedi MA, Norris SA, Micklesfield LK *et al.* (2015) Does vitamin D status track through adolescence? *Am J Clin Nutr* 102, 1025-1029.

27. Sibeko LN, Dhansay MA, Charlton KE *et al.* (2004) Full-term, peri-urban South African infants under 6 months of age are at risk for early-onset anaemia. *Public Health Nutr* 7, 813-820.

28. MAL-ED n (2017) Childhood stunting in relation to the pre- and postnatal environment during the first 2 years of life: The MAL-ED longitudinal birth cohort study. *PLoS Med* 14, e1002408.

29. Faber M & Benade AJ (2000) Factors associated with low serum retinol levels in children aged 6±24 months in a rural South African community. *Public Health Nutr* 3, 395-402.

30. Faber M, Jogessar VB Benadé AJ (2001) Nutritional status and dietary intakes of children aged 2-5 years and their caregivers in a rural South African community. *Int J Food Sci Nutr* 52, 401-411.

31. Smuts CM, Dhansay A, Faber M *et al.* (2005) Efficacy of multiple micronutrient supplementation for improving anemia, micronutrient status, and growth in South African infants. *J Nutr* 135, 653S-659S.

32. Makanjana O & Naicker A (2021) Nutritional status of children 24–60 months attending early child development centres in a semi-rural community in South Africa. *Int J Environ Res Public Health* 18, 261.

33. Motadi SA, Mbhenyane XG, Mbhatsani HV *et al.* (2015) Prevalence of iron and zinc deficiencies among preschool children ages 3 to 5 y in Vhembe district, Limpopo province, South Africa. *Nutr* 31, 452-458.

34. Carter RC, Georgieff MK, Ennis KM *et al.* (2021) Prenatal alcohol-related alterations in maternal, placental, neonatal, and infant iron homeostasis. *Am J Clin Nutr* 114, 1107-1122.

35. Rikhotso IP, Faber M, Rothman M *et al.* (2022) Nutritional status and psychomotor development in 12–18-month-old children in a post-intervention study. *S Afr J Clin Nutr* 35, 69-77.

36. Smuts CM, Matsungo TM, Malan L *et al.* (2019) Effect of small-quantity lipid-based nutrient supplements on growth, psychomotor development, iron status, and morbidity among 6- to 12-mo-old infants in South Africa: a randomized controlled trial. *Am J Clin Nutr* 109, 55-68.

37. Egal A & Oldewage-Theron W (2017) Association of micronutrients and child growth in children aged 7-15 years from Qwa-Qwa, South Africa. *S Afr J Clin Nutr* 31, 62-66.

38. Gwetu TP, Chhagan M, Craib M *et al.* (2015) Persistent and new-onset anaemia in children aged 6 - 8 years from KwaZulu-Natal Province, South Africa. *S Afr J Child Health* 9, 127.

39. Beckmann J, Lang C, du Randt R *et al.* (2021) Prevalence of Stunting and Relationship between Stunting and Associated Risk Factors with Academic Achievement and Cognitive Function: A Cross-Sectional Study with South African Primary School Children. *Int J Environ Res Public Health* 18, 4218.

40. Taljaard C, Covic NM, van Graan AE *et al.* (2013) Effects of a multi-micronutrient-fortified beverage, with and without sugar, on growth and cognition in South African schoolchildren: a randomised, double-blind, controlled intervention. *Br J Nutr* 110, 2271-2284.

41. World Health Organization (2011) *Haemoglobin concentrations for the diagnosis of anaemia and assessment of severity*. Geneva: World Health Organization.

42. Mamabolo RL & Alberts M (2014) Prevalence of anaemia and its associated factors in African children at one and three years residing in the Capricorn District of Limpopo Province, South Africa. *Curationis* 37, 1160.

43. Muriuki JM, Mentzer AJ, Webb EL *et al.* (2020) Estimating the burden of iron deficiency among African children. *BMC Med* 18, 31.

44. van der Hoeven M, Faber M, Osei J *et al.* (2016) Effect of African leafy vegetables on the micronutrient status of mildly deficient farm-school children in South Africa: a randomized controlled study. *Public Health Nutr* 19, 935-945.

45. Onabanjo OO, Jerling JC, Covic N *et al.* (2012) Association between iron status and white blood cell counts in African schoolchildren of the North-West Province, South Africa. *J Epidemiol Glob Health* 2, 103-110.

46. World Health Organization (2020) *WHO guideline on use of ferritin concentrations to assess iron status in individuals and populations.* Geneva: World Health Organization.

47. Mabapa NS MX, Jooste PL, Mamabolo RL and Amey AKA (2014) Iodine Status of Rural School children in Vhembe District of Limpopo Province, South Africa. *Curr Nutr Food Sci* 28, 2, 91-105.

48. Mabasa E, Mabapa NS, Jooste PL *et al.* (2018) Iodine status of pregnant women and children age 6 to 12 years feeding from the same food basket in Mopani district, Limpopo province, South Africa. *S Afr J Clin Nutr* 32, 76-82.

49. World Health Organization (2007) Assessment of iodine deficiency disorders and monitoring their elimination : a guide for programme managers, 3rd ed ed. Geneva: World Health Organization.

50. World Health Organization (2013) *Urinary iodine concentrations for determining iodine status in populations*. Geneva: World Health Organizations.

51. Velaphi SC, Izu A, Madhi SA *et al.* (2019) Maternal and neonatal vitamin D status at birth in black South Africans. *S Afr Med J* 109, 807-813.

52. Ncayiyana JR, Martinez L, Goddard E *et al.* (2021) Prevalence and Correlates of Vitamin D Deficiency among Young South African Infants: A Birth Cohort Study. *Nutrients* 13, 1500.

53. Poopedi MA, Norris SA Pettifor JM (2011) Factors influencing the vitamin D status of 10-year-old urban South African children. *Public health Nutr* 14, 334-339.

54. Holick MF, Binkley NC, Bischoff-Ferrari HA *et al.* (2011) Evaluation, treatment, and prevention of vitamin D deficiency: an Endocrine Society clinical practice guideline. *J Clin Endocrinol Metab* 96, 1911-1930.

55. Sarafoglou K, Rodgers J, Hietala A *et al.* (2011) Expanded newborn screening for detection of vitamin B12 deficiency. *Jama* 305, 1198-1200.

56. World Health Organization (2014) C-reactive protein concentrations as a marker of inflammation or infection for interpreting biomarkers of micronutrient status. Geneva: World Health Organization.

57. Nsibande DF & Ngandu NK (2016) Chapter 6 Child health [N Massyn, N Peer, R English, A Padarath, P Barron and C Day, editors]: Durban Health Systems Trust. https://www.hst.org.za/publications/District%20Health%20Barometers/ District%20Health%20Barometer%202015_16.pdf (accessed 16 March 2024).

58. Kongsbak K, Wahed MA, Friis H *et al.* (2006) Acute-phase protein levels, diarrhoea, Trichuris trichiura and maternal education are predictors of serum retinol: a cross-sectional study of children in a Dhaka slum, Bangladesh. *Br J Nutr* 96, 725-734.

59. Hess SY, McLain AC, Frongillo EA *et al.* (2021) Challenges for Estimating the Global Prevalence of Micronutrient Deficiencies and Related Disease Burden: A Case Study of the Global Burden of Disease Study. *Curr Dev Nutr* 5, nzab141.

60. Hess SY, McLain AC, Lescinsky H *et al.* (2021) Basis for changes in the disease burden estimates related to vitamin A and zinc deficiencies in the 2017 and 2019 Global Burden of Disease Studies. *Public Health Nutr*, 1-7.

61. van Stuijvenberg ME, Dhansay MA, Nel J *et al.* (2019) South African preschool children habitually consuming sheep liver and exposed to vitamin A supplementation and fortification have hypervitaminotic A liver stores: a cohort study. *Am J Clin Nutr* 110, 91-101.

62. Wirth JP, Petry N, Tanumihardjo SA *et al.* (2017) Vitamin A Supplementation Programs and Country-Level Evidence of Vitamin A Deficiency. *Nutrients* 9, 190.

63. Shimanda PP, Amukugo HJ Norström F (2020) Socioeconomic factors associated with anemia among children aged 6-59 months in Namibia. *J Public Health Afr* 11, 1131.

64. World Health Organization (2023) Anaemia in women and children. https://www.who.int/data/gho/data/themes/topics/anaemia_in_women_and_children (accessed 11 January 2023)

65. Ajayi OR, Matthews G, Taylor M *et al.* (2017) Factors associated with the health and cognition of 6-year-old to 8-year-old children in KwaZulu-Natal, South Africa. *Trop Med & Int Health* 22, 631-637.

66. Patel AJ, Wesley R, Leitman SF *et al.* (2013) Capillary versus venous haemoglobin determination in the assessment of healthy blood donors. *Vox sang* 104, 317-323.

67. Gedfie S, Getawa S Melku M (2022) Prevalence and Associated Factors of Iron Deficiency and Iron Deficiency Anemia Among Under-5 Children: A Systematic Review and Meta-Analysis. *Glob Pediatr Health* 9, 2333794x221110860.

68. Oldewage-Theron W & Kruger R (2017) The association between diet quality and subclinical inflammation among children aged 6-18 years in the Eastern Cape, South Africa. *Public Health Nutr* 20, 102-111.

69. World Health Organization (2014) *Serum transferrin receptor levels for the assessment of iron status and iron deficiency in populations*. Geneva: World Health Organization.

70. Tsotetsi AM, Njiro S, Katsande TC *et al.* (2013) Prevalence of gastrointestinal helminths and anthelmintic resistance on small-scale farms in Gauteng Province, South Africa. *Trop Anim Health Prod* 45, 751-761.

71. Petry N, Olofin I, Hurrell RF *et al.* (2016) The Proportion of Anemia Associated with Iron Deficiency in Low, Medium, and High Human Development Index Countries: A Systematic Analysis of National Surveys. *Nutrients* 8, 693.

72. Gwetu TP (2016) Anemia, Iron Deficiency and Diet Independently Influence Growth Patterns of School Aged Children in South Africa. *Ac J Pediatr Neonatol* 1, 555565.

73. Kondaiah P, Yaduvanshi PS, Sharp PA *et al.* (2019) Iron and Zinc Homeostasis and Interactions: Does Enteric Zinc Excretion Cross-Talk with Intestinal Iron Absorption? *Nutrients* 11, 1885.

74. Golden MH (2009) Proposed recommended nutrient densities for moderately malnourished children. *Food Nutr Bull* 30, S267-342.

75. Berhe K, Gebrearegay F Gebremariam H (2019) Prevalence and associated factors of zinc deficiency among pregnant women and children in Ethiopia: a systematic review and meta-analysis. *BMC Public Health* 19, 1663.

76. Gupta S, Brazier AKM Lowe NM (2020) Zinc deficiency in low- and middle-income countries: prevalence and approaches for mitigation. *J Hum Nutr Diet* 33, 624-643.

77. Steyn N, Nel J Labadarios D (2008) Will fortification of staple foods make a difference to the dietary intake of South African children? *S Afr J Clin Nutr* 21, 22-26.

78. Lowe NM, Fekete K Decsi T (2009) Methods of assessment of zinc status in humans: a systematic review. *Am J Clin Nutr* 89, 2040s-2051s.

79. Wieringa FT, Dijkhuizen MA, West CE *et al.* (2002) Estimation of the effect of the acute phase response on indicators of micronutrient status in Indonesian infants. *J Nutr* 132, 3061-3066.

80. Andersson M, Karumbunathan V Zimmermann MB (2012) Global iodine status in 2011 and trends over the past decade. *J Nutr* 142, 744-750.

81. Jooste PLaZ, MB (2008) Progress towards eliminating iodine deficiency in South Africa. *S Afr J Clin Nutr* 21, 8-14.

82. Charlton K, Ware LJ, Baumgartner J *et al.* (2018) How will South Africa's mandatory salt reduction policy affect its salt iodisation programme? A cross-sectional analysis from the WHO-SAGE Wave 2 Salt & Tobacco study. *BMJ open* 8, e020404.

83. Osei J, Andersson M, Reijden OV *et al.* (2016) Breast-Milk Iodine Concentrations, Iodine Status, and Thyroid Function of Breastfed Infants Aged 2-4 Months and Their Mothers Residing in a South African Township. *J Clin Res Pediatr Endocrinol* 8, 381-391.

84. UNICEF (2015) Guidance on the Monitoring of Salt Iodization Programmes and Determination of Population Iodine Status. New York: UNICEF.

85. Smuts CM BJ (2019) Are we neglecting iodine nutrition in South Africa? *S Afr J Clin Nutr* 32, 3-4.

86. Charoenngam N & Holick MF (2020) Immunologic Effects of Vitamin D on Human Health and Disease. *Nutrients* 12, 2097.

87. Mogire RM, Mutua A, Kimita W *et al.* (2020) Prevalence of vitamin D deficiency in Africa: a systematic review and meta-analysis. *The Lancet Global health* 8, e134-e142.

88. Armas LA, Dowell S, Akhter M *et al.* (2007) Ultraviolet-B radiation increases serum 25-hydroxyvitamin D levels: the effect of UVB dose and skin color. *J Am Acad Dermatol* 57, 588-593.

89. Kaferle J & Strzoda CE (2009) Evaluation of macrocytosis. *Am Fam Physician* 79, 203-208.

90. Field MS & Stover PJ (2018) Safety of folic acid. *Annals of the New York Academy of Sciences* 1414, 59-71.

91. Oliai Araghi S, Kiefte-de Jong JC, van Dijk SC *et al.* (2019) Folic Acid and Vitamin B12 Supplementation and the Risk of Cancer: Long-term Follow-up of the B Vitamins for the Prevention of Osteoporotic Fractures (B-PROOF) Trial. *Cancer Epidem Biomark* 28, 275-282.

92. Merrill RD, Burke RM, Northrop-Clewes CA *et al.* (2017) Factors associated with inflammation in preschool children and women of reproductive age: Biomarkers Reflecting Inflammation and Nutritional Determinants of Anemia (BRINDA) project. *Am J Clin Nutr* 106, 348s-358s.

93. Simbayi LC ZK, Zungu N, Moyo S, Marinda E, Jooste S, Mabaso M, Ramlagan S, North A, Van Zyl J, Mohlabane N. (2017) South African national HIV prevalence, incidence, behaviour and communication survey, 2017: towards achieving the UNAIDS 90-90-90 targets. https://hsrc.ac.za/uploads/pageContent/10779/SABSSM%20V.pdf (accessed 16 March 2024).

94. World Health Organization (2014) *C-reactive protein concentrations as a marker of inflammation or infection for interpreting biomarkers of micronutrient status*. Geneva: World Health Organization.

95. Tickell KD, Atlas HE Walson JL (2019) Environmental enteric dysfunction: a review of potential mechanisms, consequences and management strategies. *BMC Med* 17, 181.

96. Olson R, Gavin-Smith B, Ferraboschi C *et al.* (2021) Food Fortification: The Advantages, Disadvantages and Lessons from Sight and Life Programs. *Nutrients* 13.

97. Moretti D, Biebinger R, Bruins MJ *et al.* (2014) Bioavailability of iron, zinc, folic acid, and vitamin A from fortified maize. *Annals of the New York Academy of Sciences* 1312, 54-65.

98. van Jaarsveld PJ, Faber M van Stuijvenberg ME (2015) Vitamin A, Iron, and Zinc Content of Fortified Maize Meal and Bread at the Household Level in 4 Areas of South Africa. *Food Nutr Bull* 36, 315-326.

99. Steyn N, Eksteen G Senekal M (2016) Assessment of the Dietary Intake of Schoolchildren in South Africa: 15 Years after the First National Study. *Nutrients* 8.

100. Swanepoel E, Havemann-Nel L, Rothman M *et al.* (2019) Contribution of commercial infant products and fortified staple foods to nutrient intake at ages 6, 12, and 18 months in a cohort of children from a low socio-economic community in South Africa. *Matern Child Nutr* 15, e12674.

101. Micha R, Karageorgou D, Bakogianni I *et al.* (2018) Effectiveness of school food environment policies on children’s dietary behaviors: A systematic review and meta-analysis. *PLOS ONE* 13, e0194555.

102. Faber M, Laurie S, Maduna M *et al.* (2014) Is the school food environment conducive to healthy eating in poorly resourced South African schools? *Public Health Nutr* 17, 1214-1223.

103. Dannhauser A, Bester C, Joubert G *et al.* (2000) Nutritional status of preschool children in informal settlement areas near Bloemfontein, South Africa. *Public Health Nutr* 3, 303-312.

104. van Stuijvenberg ME, Schoeman SE, Lombard CJ *et al.* (2012) Serum retinol in 1-6-year-old children from a low socio-economic South African community with a high intake of liver: implications for blanket vitamin A supplementation. *Public Health Nutr* 15, 716-724.

105. Oelofse A, van Raaij JMA, Benade AJ *et al.* (2002) Disadvantaged black and coloured infants in two urban communities in the Western Cape, South Africa differ in micronutrient status. *Public Health Nutr* 5, 289-294.

106. Faber M (2007) Dietary intake and anthropometric status differ for anaemic and non-anaemic rural South African infants aged 6-12 months. *J Health Popul Nutr* 25, 285-293.

107. Heckman J, Samie A, Bessong P *et al.* (2010) Anaemia among clinically well under-fives attending a community health centre in Venda, Limpopo Province. *S Afr Med J* 100, 445-448.

108. Taljaard C, Covic N, Van Graan A *et al.* (2013) Studies since 2005 on South African primary schoolchildren suggest lower anaemia prevalence in some regions. *S Afr J Clin Nutr* 26, 168-175.

109. Samuel FO, Egal AA, Oldewage-Theron WH *et al.* (2010) Prevalence of zinc deficiency among primary school children in a poor peri-urban informal settlement in South Africa. *Health S Afr* 15, 1-6.
